# Phylogeographic Analyses of Submesophotic Snappers *Etelis coruscans* and *Etelis “marshi”* (Family Lutjanidae) Reveal Concordant Genetic Structure across the Hawaiian Archipelago

**DOI:** 10.1371/journal.pone.0091665

**Published:** 2014-04-10

**Authors:** Kimberly R. Andrews, Virginia N. Moriwake, Christie Wilcox, E. Gordon Grau, Christopher Kelley, Richard L. Pyle, Brian W. Bowen

**Affiliations:** 1 Hawai'i Institute of Marine Biology, University of Hawai'i, Kane'ohe, Hawaii, United States of America; 2 School of Biological & Biomedical Sciences, Durham University, South Road, United Kingdom; 3 Department of Oceanography, University of Hawai'i, Honolulu, Hawaii, United States of America; 4 Cell and Molecular Biology Graduate Program, University of Hawai'i, Honolulu, Hawaii, United States of America; 5 Hawai'i Undersea Research Lab, University of Hawai'i, Honolulu, Hawaii, United States of America; 6 Bernice P. Bishop Museum, Honolulu, Hawaii, United States of America; University of Otago, New Zealand

## Abstract

The Hawaiian Archipelago has become a natural laboratory for understanding genetic connectivity in marine organisms as a result of the large number of population genetics studies that have been conducted across this island chain for a wide taxonomic range of organisms. However, population genetic studies have been conducted for only two species occurring in the mesophotic or submesophotic zones (30+m) in this archipelago. To gain a greater understanding of genetic connectivity in these deepwater habitats, we investigated the genetic structure of two submesophotic fish species (occurring ∼200–360 m) in this archipelago. We surveyed 16 locations across the archipelago for submesophotic snappers *Etelis coruscans* (N = 787) and *E. “marshi”* (formerly *E. carbunculus*; N = 770) with 436–490 bp of mtDNA cytochrome *b* and 10–11 microsatellite loci. Phylogeographic analyses reveal no geographic structuring of mtDNA lineages and recent coalescence times that are typical of shallow reef fauna. Population genetic analyses reveal no overall structure across most of the archipelago, a pattern also typical of dispersive shallow fishes. However some sites in the mid-archipelago (Raita Bank to French Frigate Shoals) had significant population differentiation. This pattern of no structure between ends of the Hawaiian range, and significant structure in the middle, was previously observed in a submesophotic snapper (*Pristipomoides filamentosus*) and a submesophotic grouper (*Hyporthodus quernus*). Three of these four species also have elevated genetic diversity in the mid-archipelago. Biophysical larval dispersal models from previous studies indicate that this elevated diversity may result from larval supplement from Johnston Atoll, ∼800 km southwest of Hawaii. In this case the boundaries of stocks for fishery management cannot be defined simply in terms of geography, and fishery management in Hawaii may need to incorporate external larval supply into management plans.

## Introduction

Understanding the genetic connectivity of marine populations can provide valuable information about evolutionary mechanisms in the marine environment, as well as effective management and conservation strategies. The Hawaiian Archipelago has become a natural laboratory for understanding genetic connectivity of marine organisms, including more than 30 marine species across a wide taxonomic range (reviewed in [1]). A comparison of these species reveals at least four geographically concordant population genetic barriers, providing evidence for some commonality in population genetic structure despite the wide variety of life history characters across species. However, underlying this pattern is high variability in population structure among species, with no one species demonstrating all four of these barriers, some dispersive species showing no barriers, and many species having additional barriers. Furthermore, species with similar life history characters often do not share the same genetic barriers, indicating that any single species cannot be used as an exemplar for dispersal in the Hawaiian Archipelago.

**Figure 1:**
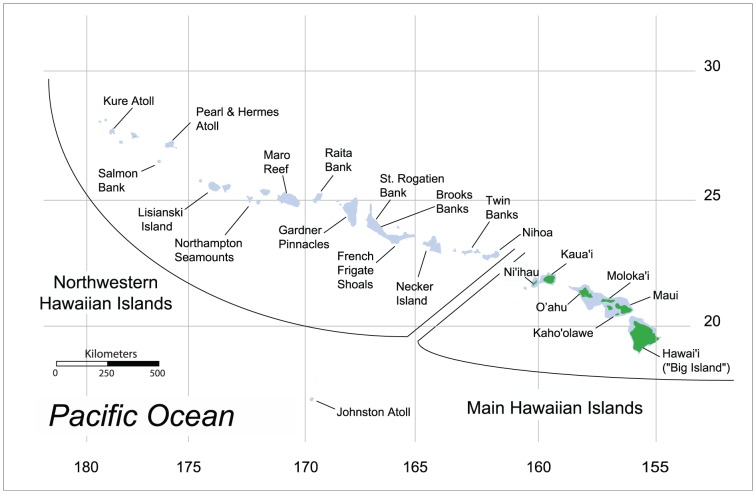
Map of the Hawaiian Archipelago and Johnston Atoll showing sampling locations and geographic division between the Northwestern Hawaiian Islands and the Main Hawaiian Islands.

The population genetic studies conducted thus far in the Hawaiian Archipelago have included only two species which occur at the lower margin of photosynthetic activity in the mesophotic and submesophotic zones, the Hawaiian Grouper, called “Hapu'upu'u” in Hawai'i (*Hyporthodus quernus*, previously *Epinephelus quernus*) [2], [3] and the Crimson Jobfish, called “Opakapaka” in Hawai'i (*Pristipomoides filamentosus*) [4]. These studies found interesting patterns of genetic structure: both species showed evidence of high connectivity across the Hawaiian Archipelago, but both species also showed evidence for weak but significant genetic divergence at several sites in the middle of the archipelago. The Hawaiian grouper also had elevated genetic diversity at a mitochondrial DNA (mtDNA) marker in the mid-archipelago. Bio-physical simulations of larval dispersal indicated that this genetic distinctiveness of the mid-archipelago may result from connectivity between the mid-archipelago and Johnston Atoll, the closest landmass to the Hawaiian Archipelago (Figure 1) [3], [5]. These simulations also indicated that directional dispersal from the outer edges of the archipelago toward the mid-archipelago may contribute to the elevated mtDNA diversity [3].

The pattern of genetic structure and high genetic diversity in the mid-archipelago has not been observed in other fish species studied in Hawai'i thus far. These studies include 15 shallow-water reef fish species, most of which exhibit high connectivity across most or all of the archipelago, with the exception of one damselfish (*Dascyllus albisella*) that shows strong genetic divergence among all islands sampled across the archipelago, and one surgeonfish (*Zebrasoma flavescens*) that shows low genetic structure [6]–[13]. The pattern of genetic structure and high diversity in the mid-archipelago has not been observed for invertebrate species either (e.g. [14], [15]).

To test the hypothesis that the pattern of genetic structure and high diversity in the mid-archipelago is consistent for other deepwater fishes, we conducted population genetic analyses of two submesophotic fishes which occur across the archipelago: the Flame Snapper, called “Onaga” in Hawai'i (*Etelis coruscans*) and the Ruby Snapper, called “Ehu” in Hawai'i (*Etelis “marshi*”, currently being revised from *Etelis carbunculus*, Andrews *et al.* in prep). Adult *E. coruscans* and *E. marshi* typically occur at deeper depths (200–320 m for *E. coruscans*; 200–360 m for *E. marshi*) than adult *P. filamentosus* and *H. quernus* (80–240 m for *P. filamentosus*, and 120–280 m for *H. quernus*; but adult *H. quernus* occur at <10 m depth in the far northwestern atolls of the Hawaiian Archipelago) [16]–[18]. However, juveniles of these species are sometimes found at shallower, mesophotic depths [16], [19], [20].

The adult habitat of all four of these species is similar in that it consists of hard substrate with high structural complexity and/or high relief [21]. Despite their similar habitat, however, these species have different habitat use, and these differences may influence adult dispersal and genetic connectivity. Adult *E. marshi* and *H. quernus* are more closely associated with the bottom substrate including crevasses and under ledges (C.K. pers. obs., [16]) and therefore may exhibit greater genetic structure than other species due to lower dispersal by juveniles and adults. The close association with the bottom for these species may be due to partitioning of feeding niches between benthos and water column, a greater need for protection due to small adult size (*E. marshi*), or a suspected territorial, haremic social structure (*H. quernus*). In contrast, *E. coruscans* and *P. filamentosus* are more pelagic feeders that usually occur 2–50 meters above the bottom.

To investigate the population genetic structure of *E. coruscans* and *E. marshi*, we used mtDNA cytochrome *b* (cyt*b*) sequences and 10–11 microsatellite loci from 16 sample sites across the archipelago (Figure 1, Table 1, Table 2). Our study was initially motivated by fishery management concerns for these popular food fishes subject to commercial and recreational catches. However, our study is also one of the first to examine the population genetic structure of a submesophotic species, and thus we aimed to explore the influence of this unique habitat on the evolution of fishes compared to shallow-water habitats. For example, deepwater habitat occupied by submesophotic fishes may have lower water movement than shallow habitat due to the lack of Ekman surface transport and waves, which could result in less dispersive larvae and stronger genetic structure. We tested this hypothesis through qualitative comparisons of the population genetic structure of *E. coruscans* and *E. marshi* with the population genetic structure reported in previous studies for two other submesophotic fishes (*P. filamentosus* and *H. quernus*) and shallow-water fishes in the Hawaiian Archipelago. Additionally, submesophotic habitats may lie below the zone disrupted by sea level changes during glaciations, and thus communities in this deepwater habitat may be older and more stable than their shallow counterparts. We tested this hypothesis by estimating mtDNA coalescent times for *E. coruscans* and *E. marshi*, and comparing these values with previously published mtDNA coalescent times from shallow-water fishes.

**Table 1:** Sample sizes, diversity indices, and sampling years for mtDNA cytochrome b (cyt*b*) and microsatellite loci across sample sites in the Hawaiian Archipelago for *Etelis coruscans*. *h*, haplotype diversity; π, nucleotide diversity; *A*_R_ allele richness; *H_o_*, observed heterozygosity; *H_e_*, expected heterozygosity.

			cyt*b*	Microsatellites				
Location	*n*	*h*	*π*	*n*	*A_R_*	*H_o_*	*H_e_*	Sampling years
Pearl & Hermes	12	0.67+/−0.14	0.0021+/−0.0017	11	4.56	0.712	0.767	1998
Lisianski	34	0.75+/−0.06	0.0024+/−0.0017	29	4.44	0.691	0.752	1998(17), 2006(17)
Maro Reef	44	0.67+/−0.05	0.0019+/−0.0015	41	4.50	0.722	0.765	1998(42), 2006(2)
Raita	26	0.74+/−0.09	0.0024+/−0.0018	18	4.17	0.668	0.726	2005–06
Gardner	104	0.78+/−0.03	0.0027+/−0.0019	84	4.40	0.643	0.748	2006
St. Rogatien	23	0.84+/−0.07	0.0039+/−0.0026	23	4.42	0.703	0.744	2005
Brooks Banks	76	0.69+/−0.04	0.0020+/−0.0015	67	4.47	0.682	0.749	2005–06
Twin Banks	14	0.75+/−0.11	0.0024+/−0.0018	14	4.37	0.704	0.749	1998
Nihoa	43	0.79+/−0.05	0.0028+/−0.0019	39	4.26	0.736	0.735	2007
Ni'ihau	51	0.76+/−0.04	0.0024+/−0.0018	50	4.43	0.719	0.760	2005–06
Kaua'i	69	0.76+/−0.04	0.0028+/−0.0019	56	4.60	0.679	0.762	1998
O'ahu	55	0.73+/−0.04	0.0023+/−0.0017	47	4.48	0.726	0.762	1997–98(37), 2006–07(18),
Moloka'i	92	0.78+/−0.03	0.0026+/−0.0018	92	4.45	0.697	0.749	1998
Maui	50	0.73+/−0.05	0.0021+/−0.0016	45	4.29	0.722	0.732	2006–2007
Kaho'olawe	11	0.78+/−0.09	0.0022+/−0.0018	9	4.62	0.739	0.813	2006
Big Island	83	0.76+/−0.04	0.0025+/−0.0018	83	4.42	0.701	0.752	1998
Total	787			708				

For geographic locations with sampling intervals >1 year apart, the number of specimens sampled per interval is given in parentheses.

**Table 2:** Sample sizes, diversity indices, and sampling years for mtDNA cytochrome b (cyt*b*) and microsatellite loci across sample sites in the Hawaiian Archipelago for *Etelis marshi*. *h*, haplotype diversity; π, nucleotide diversity; *A*_R_ allele richness; *H_o_*, observed heterozygosity; *H_e_*, expected heterozygosity.

			cyt*b*	Microsatellites				
Location	*n*	*h*	*π*	*n*	*A_R_*	*H_o_*	*H_e_*	Sampling years
Salmon Bank	11	0	0	11	4.00	0.631	0.664	2006
Lisianski	12	0	0	10	2.94	0.600	0.627	2006–07
Northampton	81	0.23+/−0.06	0.00056+/−0.00071	83	2.85	0.572	0.604	1998(68), 2006–07(15)
Maro Reef	46	0.21+/−0.08	0.00060+/−0.00075	47	2.87	0.564	0.617	2006–07
Raita	46	0.25+/−0.08	0.00080+/−0.00089	33	2.92	0.580	0.616	2005–06
Gardner	31	0.49+/−0.10	0.00141+/−0.00128	32	2.83	0.563	0.615	2006
St. Rogatien	31	0.19+/−0.09	0.00045+/−0.00064	30	3.03	0.589	0.624	2006
Brooks Banks	10	0.38+/−0.18	0.00185+/−0.00164	11	3.07	0.624	0.647	2005–06
Necker	64	0.24+/−0.07	0.00064+/−0.00077	64	2.99	0.552	0.633	1998(21), 2003(25), 2006–07(19)
Twin Banks	71	0.24+/−0.07	0.00065+/−0.00078	74	2.95	0.558	0.624	1998
Ni'ihau	76	0.22+/−0.06	0.00060+/−0.00074	78	2.85	0.581	0.607	1998(41), 2006(37)
Kaua'i	44	0.21+/−0.08	0.00051+/−0.00068	44	2.96	0.593	0.622	1998
O'ahu	34	0.17+/−0.09	0.00054+/−0.00071	37	2.85	0.604	0.609	1998(18), 2006–07(16)
Moloka'i	64	0.15+/−0.06	0.00043+/−0.00062	67	2.90	0.563	0.614	1998(63), 2005–07(4)
Maui	74	0.25+/−0.07	0.00073+/−0.00084	74	2.83	0.583	0.610	2006
Big Island	73	0.23+/−0.07	0.00056+/−0.00072	75	2.84	0.586	0.607	1998
Total	768			770				

For geographic locations with sampling intervals >1 year apart, the number of specimens sampled per interval is given in parentheses.

## Methods

Specimens consisted of fin or muscle tissue collected throughout the Hawaiian Archipelago from 16 locations each for *E. coruscans* (N = 787) (Table 1, Figure 1) and *E. marshi* (N = 770) (Table 2, Figure 1). Specimens were collected by commercial fishers who recorded GPS coordinates for each sampling location and stored the specimens frozen or in salt-saturated DMSO buffer [22]. Most fishing activity for these species takes place at a depth of 200–300 m, with distances from shore ranging from a few hundred meters to as far as 65 km. Specimens were collected in 1997–1998 or 2003–2007 (Table 1, Table 2). Genomic DNA was extracted using a phenol chloroform method [23], DNeasy extraction kits (Qiagen, Valencia, CA, USA), or the Hotshot method [24].

### MtDNA sequencing

PCRs amplified portions of mtDNA cytochrome *b* gene for both species. Primers used for *E. coruscans* were Cyb-05 L15020 (GCCAACGGCGCATCCTTCTTCTT [25]) and Cyb-07 H15573 (AATAGGAAGTATCATTCGGGTTTGATG [26]), amplifying approximately 560 bp. Primers used for *E. marshi* were designed for this study: EhucybF (TCAGTCGCACACATCTGCCG) and EhucybR (AGTGCAACAAGGACGGCTGC) and amplified 524 bp. PCRs were performed in 15 µl volumes containing 1× MangoMix (Bioline, Taunton MA, USA) and 0.2 µM each primer. For *E. coruscans*, cycle conditions were as follows: 94°C for 1 min; 35 cycles of 94°C for 30 sec, annealing temperature 55°C for 45 sec; and 72°C for 30 sec; and a final 72°C extension for 10 min. Cycle conditions for *E. marshi* were the same with the exception of the annealing temperature step, which was 68°C for 30 sec. To clean PCR products, 7.5 units of Exonuclease I and 0.75 units of FastAP alkaline phosphatase (Fermentas Life Sciences, Ontario, Canada) were added to 7.5 µl of PCR product, and aliquots were incubated at 37°C for 60 minutes and 85°C for 15 minutes. PCR products were then sequenced in one direction with an ABI3730 automated sequencer (Applied Biosystems, Foster City, CA). Questionable or low quality sequences were resequenced in the forward direction. Sequences were edited and aligned using GENEIOUS PRO 5.6.2 (Biomatters, LTD, Auckland, NZ).

### Microsatellite genotyping

A total of 16 variable microsatellite loci were developed using genomic DNA extracted from *E. coruscans* and *E. marshi* through a procedure including restriction enzyme digestion, microsatellite enrichment, cloning of DNA fragments, and DNA sequencing of clones (*sensu* [27]) (Table 3). Five of these loci were PCR-amplified for both species. An additional five loci were amplified for *E. coruscans*, resulting in a total of 10 loci for this species; and an additional six loci were amplified for *E. marshi*, resulting in a total of 11 loci for this species (Table 3). Multiplex PCRs were carried out using fluorescent dye-labeled forward primers and Qiagen Type-It Microsatellite PCR Kits, with three or four loci included per multiplex reaction, and following reaction conditions recommended by Qiagen. PCR products were separated on ABI 3730XL or ABI 3130XL genetic analyzers, with all PCR products from each primer set run exclusively on only one of these two analyzers to avoid bias in fragment size assignments. Fragment sizes were scored using GENEMAPPER 4.0 (Applied Biosystems).

**Table 3:** Characteristics of 16 microsatellite loci developed for *Etelis coruscans* and *Etelis marshi*, including number of alleles (*k*), observed heterozygosity (*H_o_*), and expected heterozygosity (*H_e_*) across the Hawaiian Archipelago for each locus.

				*E. coruscans*				*E. marshi*			
Locus	Fwd primer (5′-3′)	Rev primer (5′-3′)	Repeat motif	Size range	k	Ho	He	Size range	k	Ho	He
EtelisE20O2	GGCCCCATATTAACTTCTAGTGC	TTGTTCATCTCACAAAACATTGG	TG	183–231	36	0.844	0.864				
EtelisE21O2	GGAGCAAATCAACATTTCAGG	ACCAGGGAACAGGTGAAGG	TG	191–203	9	0.424	0.429				
EtelisI11O2	GGCATGTCAAGAAAAGTTTGG	AAAGAAAAGCGATGGAAGACC	CA	172–196	13	0.675	0.758				
EtelisO17O1	CTTCTGTGTTATCAATCTCAATCTGC	TCCCCTTTTTCCTCTCATCC	GGAT	162–190	8	0.496	0.519				
EtelisO22O2	CAATTTAAAAGCCCAACAGACC	GCACTGCATCATGTTTTCTCC	CA	187–221	32	0.717	0.906				
EtelisC19O2	GGACTCCAGGCCGATAGC	CCTGCCAGTAGGAACAAAGC	CA	157–205	23	0.852	0.893	158–160	2	0.457	0.468
EtelisE2E2	TTTATTGACCAGCTCTAACAGAGG	GTGTGATTTCCGATGAAACG	CA	262–296	18	0.761	0.758	282–413	22	0.777	0.785
EtelisG8E1	GAGCTGGAGCAACTGAATCC	TTATTCACACGCAGCATTAGC	GGAT	150–186	10	0.720	0.730	154–198	11	0.347	0.741
EtelisG23O1	GTGAATCACAAAGATCCTTCAGC	TCTCCACTCGGCATGAGC	ACAC	166–217	18	0.579	0.798	169–248	27	0.855	0.848
EtelisM15E1	AACAGAGTGGGAGGGAAGG	GACCCCTGGGAGTCAATG	GA	101–153	28	0.902	0.910	105–125	8	0.579	0.538
EtelisA14E2	AATCACTTTAATATTGCGATACTTGC	GAACATGATCACGATACAACAGC	TTG					257–263	3	0.039	0.118
EtelisC1E2	TCACGATGGTCACTTCATGC	CAGAATCACCAGAGGGAAGC	CA					274–336	23	0.725	0.710
EtelisE20O1	CAATGGAGCTGGTTGATCG	GAAGGGATGAACAAGTGATGG	ATCC					133–166	9	0.547	0.547
EtelisE6E1	TCACACACAAACACACACAGC	CGCTAGCCCTCTTTCTTCC	AAGG					216–272	8	0.709	0.699
EtelisI3O1	AATGGCTTGTGTATCCTGTGC	TTCATCCATCCAGCAGTAACC	GGAT					147–188	10	0.679	0.714
EtelisP15E1	TGTAGGTTTAAAGAACTGGCTCTG	AGCTGTTTTGGCATATTTTGTC	GGAT					192–220	8	0.610	0.598

MSTOOLS 3.1 [28] was used to identify identical genotypes to confirm that no individual fish specimens were present more than once in the dataset. Each microsatellite locus was tested for departures from Hardy Weinberg equilibrium and linkage equilibrium using ARLEQUIN 3.11 [29]. Each locus was also tested for null alleles using MICRO-CHECKER 2.2.0.3 [30]. Null allele frequencies were estimated using FREENA [31].

### Genetic Diversity and Population Structure

For the mtDNA, nucleotide (π) and haplotype (*h*) diversities were obtained with ARLEQUIN. The nucleotide substitution models used to calculate genetic distance were Tamura Nei+gamma = 0.403 [32] for *E. coruscans* and Tamura Nei for *E. marshi*; these were the best-fit models chosen using the AIC method in jModeltest 0.1.1 [33]. For microsatellites, observed heterozygosity, expected heterozygosity, allele richness, and total number of alleles were calculated for each locus using FSTAT 2.9.3.2 [34] and ARLEQUIN.

For mtDNA, genetic similarity between geographic locations was investigated with median-joining haplotype networks for both *E. coruscans* and *E. marshi* using NETWORK 4.6.1.0 [35]. The maximum parsimony (MP) option was used to remove unnecessary median vectors and links [35].

Genetic similarity between geographic locations was further investigated by calculating population pairwise *Φ*_ST_ values for mtDNA and *F*_ST_ for microsatellites with ARLEQUIN. For mtDNA analyses, genetic distance was calculated using nucleotide substitution models as described above. For both genetic assays, significance of pairwise values was tested using 20,000 permutations. For microsatellites, the influence of each individual locus on multi-locus pairwise *F*_ST_ values was investigated by removing one locus at a time; and the influence of null alleles on *F*_ST_ analyses was investigated by estimating global *F*_ST_ values with and without correcting for null alleles using the ENA method as implemented in FREENA [31].

To investigate the influence of temporal sampling on the estimation of allele frequencies and inference of genetic structure, we conducted pairwise *F*_ST_ analyses comparing samples collected more than three years apart within a given geographic location. These analyses were conducted for any geographic sample location containing more than 10 individuals each per temporal period (i.e. Lisianski and O'ahu for *E. coruscans*; and Northampton, Necker, and Ni'ihau for *E. marshi*).

Population genetic structure was further analyzed using Bayesian clustering analyses implemented in STRUCTURE 2.3.3 [36] for microsatellite loci, using the admixture and correlated allele frequency models. The burn-in length was set at 10^5^ steps, followed by 10^6^ steps. Analyses were run five times for each of *K* = 1 to *K* = 16 to test for consistency of estimates of *P(X|K)*, where *K* is the number of clusters. Bayesian clustering analyses were conducted both with and without use of sampling location as a prior; using sampling location as a prior has been shown to recover population structure at lower levels of divergence, without bias towards assigning structure when it is not present [37].

Relationships between geographic distance and genetic divergence were investigated using GENEPOP 4.0.10 [38], [39] for both mtDNA and microsatellite data. Mantel tests (10,000 permutations) and Spearman Rank correlation tests were used to examine correlations between geographic distance *vs.* genetic divergence (*Φ*_ST_/(1−*Φ*_ST_) [40]). Geographic distance was calculated as the shortest great-circle distance between the approximate centers of sampling sites.

To resolve evolutionary histories, Tajima's *D* and Fu's *F*_s_ tests [41] for departure from mutation-drift equilibrium were conducted with ARLEQUIN using mtDNA cyt*b* sequences. Large negative values of Tajima's *D* and Fu's *F*_s_ are expected to occur if populations have experienced selection or recent expansions [42]–[44]. For Tajima's *D*, a significant positive value is expected if populations are admixed or experiencing diversifying selection. Fu's *F*_s_ is expected to have greater power than Tajima's *D* for detecting population expansions [45]. To estimate the age, historic female effective size, and post-expansion female effective size of Hawaiian populations, a coalescence analysis was performed with ARLEQUIN, assuming a cyt*b* rate of 2% per million years between lineages (1% within lineages) calibrated with other marine fishes [46], [47] Generation time was estimated at 10 years based on available life history data: recent evidence indicates that female *E. marshi* in the Hawaiian Archipelago may mature at two years of age (E. DeMartini, pers. comm.), and the maximum age for *E. coruscans* and *E. marshi* in the Hawaiian Archipelago is estimated at 13 years [48] (although this may be an underestimate of the maximum age given that *E. carbunculus* in the Indian Ocean have lifespans of at least 35 years [49]). Because both mutation rate and generation time are not validated for *Etelis* species, corresponding estimates of effective population size and population age should be interpreted with caution. However, they should suffice to provide first order approximations.

## Results

Microsatellite allele fragment lengths and cytb GenBank Accession No.'s (KF920464 to KF920552) are reported for each individual specimen in Table S1 for *E. coruscans* and in Table S2 for *E. marshi*.

Identification of matching microsatellite and mtDNA genotypes indicated that two *E. marshi* specimens were present twice in the dataset: one specimen was present twice in the Maro Reef sample, and the other present twice in the Necker sample. One of each duplicate was removed from the dataset for subsequent mtDNA and microsatellite analyses. No *E. coruscans* specimens were present more than once in the dataset.

### Microsatellite quality control

For *E. coruscans*, three loci showed evidence of deviation from HWE after Bonferroni correction, including locus EtelisO22O2 (seven geographic locations), EtelisG23O1 (four locations), and EtelisC19O2 (one location). These loci also exhibited an excess of homozygotes at two or more locations (Etelis O22O2 – 11 locations, EtelisG23O1 – 11 locations, Etelis C19O2 – 2 locations), indicating that deviations from HWE may result from null alleles. Three additional loci exhibited evidence of homozygote excess at one or more locations: EtelisI11O2 (five locations), EtelisM15E1 (one location), and EtelisG8E1 (one location). FREENA indicated a relatively low frequency of null alleles across loci and populations (range: 0.00–0.25, mean: 0.03).

For *E. marshi*, only one locus (EtelisG8E1) showed evidence of deviation from HWE after Bonferroni correction; this locus deviated from HWE at 12 geographic locations. This locus also exhibited an excess of homozygotes at 11 locations, indicating that null alleles may be responsible for deviations from HWE. Two additional loci exhibited evidence for homozygote excess: EtelisA14E2 (six locations) and EtelisE20O1 (one location). Loci which exhibited evidence for homozygote excess generally had higher null allele frequency estimates across populations (EtelisG8E1 range: 0.13–0.32, mean: 0.22; EtelisA14E2: 0.00–0.23, mean: 0.10; EtelisE20O1: 0.00–0.20, mean: 0.01) than other loci (0.00–0.18, mean: 0.01).

No loci showed evidence for linkage disequilibrium for either *E. coruscans* or *E. marshi* after Bonferroni correction. Global *F*_ST_ values calculated with and without correcting for null alleles had overlapping 95% confidence intervals for both species. Additionally, removing one locus at a time had little impact on pairwise *F*_ST_ results for either species. For *E. coruscans*, average deviations between all pairwise *F*_ST_ values calculated using all loci versus all except one locus ranged from 0.0021 (when removing EtelisE2E2) to 0.0059 (when removing EtelisE20O2). For *E. marshi*, average deviations between all pairwise *F*_ST_ values calculated using all loci versus all except one locus ranged from 0.0015 (when removing EtelisA14E2) to 0.0133 (when removing EtelisE6E1). For each species, the greatest deviations in average *F*_ST_ occurred with the removal of a locus that showed no evidence of null alleles. Below we report results from analyses using all loci.

### Genetic diversity and population structure

Editing of cyt*b* DNA sequence data resulted in a 490 bp fragment for *E. coruscans* and a 436 bp fragment for *E. marshi*. Diversity values (*h* and π) were consistently higher for *E. coruscans* than E. *marshi.* For *E. coruscans*, *h* ranged from 0.67 to 0.84 and π ranged from 0.0019–0.0039 (Table 1), with the highest values for both *h* and π occurring at St. Rogatien in the middle of the archipelago (Figure 1). For *E. marshi*, two locations (Salmon Bank and Lisianski) had zero diversity (*h* = π = 0); however, this low diversity may be related to the small sample sizes at these locations (*n* = 11 and *n* = 12) (Table 2). For *E. marshi* at all other geographic locations, *h* ranged from 0.15 to 0.49 and π ranged from 0.00043 to 0.00185 (Table 2), with the highest value for *h* occurring at Gardner and for π occurring at Brooks Banks, and the second highest value for π occurring at Gardner. Higher diversity values for *E. coruscans* than *E. marshi* were not driven by the longer cyt*b* fragment resolved for *E. coruscans*; when diversity values were calculated using only the region of overlap between cyt*b* sequences for the two species (379 bp), *E. coruscans* still had consistently higher diversity values than *E. marshi*, except at Gardner, where haplotype diversity was slightly higher for *E. marshi* (data not shown).

For microsatellites, observed heterozygosity across the Hawaiian Archipelago for *E. coruscans* ranged from 0.643 to 0.739, and expected heterozygosity ranged from 0.726 to 0.813 (Table 1). For *E. marshi*, observed heterozygosity ranged from 0.552 to 0.631, and expected heterozygosity ranged from 0.604 to 0.664 (Table 2).

**Figure 2:**
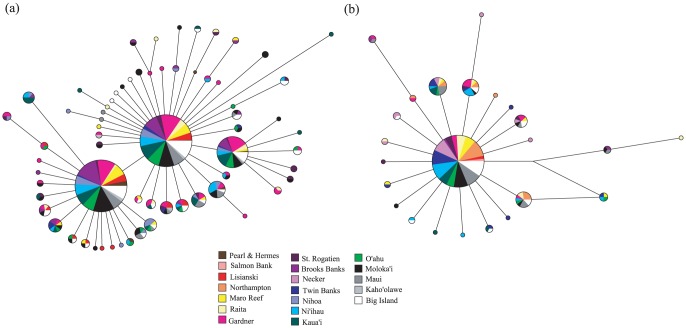
Median-joining network of cytochrome *b* haplotypes for (a) *Etelis coruscans* and (b) *Etelis marshi* obtained from the program NETWORK 4.6.1.0 (Bandelt *et al.* 1999). Each circle represents a haplotype; circle sizes are proportional to the frequency of haplotypes; and line lengths are proportional to the number of mutational steps between haplotype sequences.

For *E. coruscans*, NETWORK analyses resulted in multiple maximum parsimony networks, all of which were similar in structure, with one of these reported here (Figure 2a). For *E. marshi*, NETWORK analyses produced only one maximum parsimony network (Figure 2b). Networks for both species showed overall low genetic divergence between haplotypes, with no more than 5 mutations separating any two haplotypes. For both *E. coruscans* and *E. marshi*, haplotype networks revealed no obvious segregation of haplotypes by geographic location.

**Figure 3:**
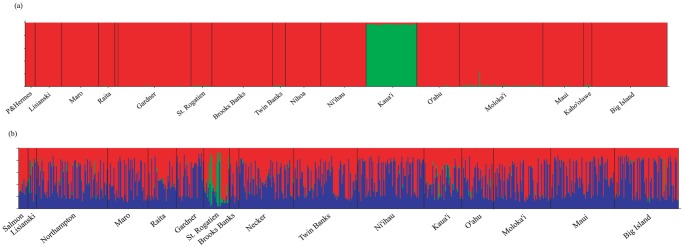
Bayesian clustering analysis results obtained with the program STRUCTURE 2.3.3 (Pritchard 2000) using sample location as a prior for (a) *Etelis coruscans*, *K* = 2 and (b) *Etelis marshi*, *K* = 3.

For *E. coruscans*, pairwise *Φ*_ST_ analyses for mtDNA revealed significant divergence for 20 out of 120 pairwise comparisons, with all except one of these comparisons involving locations in the mid-archipelago (Raita through Brooks Banks) (Table 4). For microsatellite loci, only three pairwise *F*_ST_ comparisons revealed significant divergence (Maro Reef. vs. Molokai, Twin Banks vs. Molokai, and Nihoa vs. Kahoolawe) (Table 4). For Bayesian clustering analyses without using sampling location as a prior, average posterior probabilities were consistently highest at *K* = 1. When sampling location was used as a prior, average posterior probabilities were consistently highest at *K* = 3; however, visual inspection of the output from all runs of *K* = 3 indicated only two consistent population clusters: (1) Kaua'i and (2) all other locations (Figure 3a).

**Table 4:** Pairwise population genetic divergence for mtDNA cyt*b* sequences (*Φ*_ST_, below diagonal) and microsatellites (*F*_ST_, above diagonal) across the Hawaiian Archipelago for *Etelis coruscans*.

	1	2	3	4	5	6	7	8	9	10	11	12	13	14	15	16
**1. Prl & Hermes**		0.002	0.000	0.003	−0.004	0.001	−0.001	−0.002	−0.036	−0.003	−0.030	−0.010	0.007	0.000	−0.008	−0.005
**2. Lisianski**	0.007		−0.009	0.002	−0.009	−0.009	−0.007	−0.009	−0.039	−0.015	−0.001	−0.011	0.003	−0.003	−0.006	−0.010
**3. Maro Reef**	0.028	−0.008		−0.010	−0.004	0.005	−0.002	0.001	−0.044	−0.003	−0.016	−0.022	**0.008** **	−0.006	−0.014	0.000
**4. Raita**	**0.166** **	**0.088** **	**0.076** **		−0.013	−0.010	−0.003	−0.009	−0.010	−0.019	0.003	−0.001	−0.016	−0.001	−0.007	−0.024
**5. Gardner**	**0.064** *	**0.027** *	0.016	0.003		−0.003	−0.001	−0.010	−0.005	−0.012	−0.034	−0.018	−0.009	−0.007	0.005	−0.019
**6. Rogatien**	**0.069** *	0.037	0.033	−0.013	−0.009		0.000	0.007	−0.033	−0.003	−0.033	−0.023	−0.002	−0.009	−0.007	−0.004
**7. Brooks Banks**	−0.003	−0.002	−0.005	**0.099** **	**0.029** **	**0.051** *		−0.011	−0.018	−0.009	−0.031	−0.008	−0.002	0.000	0.006	−0.011
**8. Twin Banks**	0.091	0.015	0.007	0.001	−0.013	−0.020	0.028		−0.063	0.003	−0.018	−0.020	**0.011** *	−0.002	−0.024	0.005
**9. Nihoa**	−0.010	−0.003	−0.006	**0.081** **	**0.027** *	**0.035** *	−0.002	0.022		−0.059	−0.064	−0.037	−0.027	−0.017	**0.013** *	−0.053
**10. Ni'ihau**	0.023	0.007	−0.002	**0.043** *	0.008	0.021	0.008	−0.001	0.001		−0.025	−0.027	0.002	−0.014	−0.028	0.000
**11. Kaua'i**	0.049	0.022	0.008	0.020	0.000	0.001	0.017	−0.008	0.015	0.001		−0.023	−0.014	−0.007	−0.030	−0.027
**12. O'ahu**	0.028	0.002	−0.006	**0.041** *	−0.001	0.007	−0.001	−0.007	0.001	−0.002	−0.003		−0.015	0.000	−0.024	−0.023
**13. Moloka'i**	0.004	−0.002	−0.007	**0.056** **	**0.014** *	0.026	−0.004	0.002	−0.001	−0.002	0.010	−0.006		−0.006	−0.014	−0.001
**14. Maui**	0.067	0.012	−0.001	**0.036** *	0.007	0.017	**0.025** *	−0.003	0.009	−0.003	0.006	0.004	0.005		−0.002	−0.020
**15. Kaho'olawe**	0.032	−0.026	−0.025	0.000	−0.034	−0.038	−0.013	−0.047	−0.018	−0.027	−0.031	−0.036	−0.030	−0.027		−0.028
**16. Big Island**	**0.063** *	0.016	0.004	0.013	−0.004	0.003	**0.020** *	−0.010	0.019	0.002	−0.002	−0.006	0.006	−0.001	−0.033	

Values in bold are significant:

*p<0.05,

**p<0.01.

For *E. marshi*, pairwise *Φ*_ST_ analyses for mtDNA revealed significant divergence for 9 out of 120 pairwise comparisons, and each of these comparisons involved Gardner (adjacent to St. Rogatien) (Table 5). Pairwise *F*_ST_ comparisons for microsatellite loci revealed significant divergence for 4 out of 120 pairwise comparisons, with all but one of these comparisons (Northampton vs. Niihau) involving a location in the mid-archipelago (Gardner or Raita) (Table 5). For Bayesian clustering analyses that did not use sampling location as a prior, average posterior probabilities were consistently highest at *K* = 1. When sampling location was used as a prior, average posterior probabilities were consistently highest at *K* = 2, and visual inspection of the output indicated two consistent population clusters: (1) St. Rogatien in the middle of the archipelago, and (2) all other locations (Figure 3b).

**Table 5:** Pairwise population genetic divergence for mtDNA cyt*b* sequences (*Φ*_ST_, below diagonal) and microsatellites (*F*_ST_, above diagonal) across the Hawaiian Archipelago for *Etelis marshi*.

	1	2	3	4	5	6	7	8	9	10	11	12	13	14	15	16
**1. Salmon Bank**		−0.215	−0.217	−0.198	−0.051	−0.213	−0.051	−0.140	−0.170	−0.208	−0.210	−0.246	−0.220	−0.195	−0.194	−0.218
**2. Lisianski**	0.000		−0.025	0.007	−0.042	−0.005	−0.052	−0.044	0.010	−0.021	−0.032	−0.025	−0.008	−0.018	0.005	−0.008
**3. Northampton**	−0.035	−0.030		−0.011	−0.076	−0.001	−0.080	−0.037	−0.026	0.000	**0.004** *	−0.004	−0.003	0.003	−0.015	−0.001
**4. Maro Reef**	−0.045	−0.041	−0.007		−0.049	−0.012	−0.048	−0.042	−0.002	−0.011	−0.008	−0.015	0.001	−0.005	−0.001	−0.004
**5. Raita**	−0.041	−0.037	−0.009	−0.013		−0.079	**0.010** *	−0.070	−0.035	−0.073	−0.073	−0.088	−0.067	−0.069	−0.041	−0.071
**6. Gardner**	−0.003	0.002	**0.034** *	0.022	**0.024** *		−0.071	−0.033	−0.021	0.001	**0.008** *	0.001	0.002	**0.008** *	−0.011	−0.001
**7. St. Rogatien**	−0.042	−0.037	0.002	−0.014	−0.004	0.011		−0.052	−0.032	−0.073	−0.072	−0.092	−0.079	−0.071	−0.051	−0.071
**8. Brooks Banks**	0.009	0.018	0.077	0.047	0.024	0.007	0.059		−0.044	−0.037	−0.040	−0.052	−0.043	−0.039	−0.029	−0.039
**9. Necker**	−0.042	−0.038	0.005	−0.007	−0.002	**0.037** **	−0.007	0.064		−0.029	−0.025	−0.029	−0.016	−0.023	−0.002	−0.020
**10. Twin Banks**	−0.041	−0.037	−0.002	−0.012	−0.006	**0.043** **	−0.002	0.059	−0.003		0.001	−0.009	−0.001	−0.001	−0.014	−0.003
**11. Ni'ihau**	−0.035	−0.031	−0.004	−0.008	−0.007	0.021	−0.007	0.070	0.004	0.000		−0.002	0.002	−0.001	−0.010	−0.002
**12. Kaua'i**	−0.026	−0.021	0.009	0.001	0.004	**0.055** ***	0.014	0.075	−0.001	−0.009	0.015		−0.003	−0.008	−0.012	−0.007
**13. O'ahu**	−0.032	−0.027	−0.013	−0.014	−0.018	**0.037** *	0.008	0.044	0.003	−0.009	−0.002	0.004		0.000	−0.006	−0.002
**14. Moloka'i**	−0.046	−0.042	0.000	−0.012	−0.008	**0.040** **	−0.010	0.070	0.000	−0.001	−0.002	0.016	−0.003		−0.006	−0.002
**15. Maui**	−0.034	−0.030	−0.004	−0.007	−0.008	**0.039** **	0.005	0.036	0.002	−0.005	0.007	−0.005	−0.015	0.003		−0.010
**16. Big Island**	−0.041	−0.037	−0.002	−0.010	−0.004	**0.026** *	−0.013	0.077	−0.002	0.002	−0.004	0.015	−0.001	−0.007	0.005	

Values in bold are significant:

*p<0.05,

**p<0.01,

***p<0.001.

For both species, analyses comparing samples collected during different time periods within a given geographic location resulted in no significant pairwise *Φ*_ST_ or *F*_ST_ values for any location or any genetic marker (*P*>0.05, data not shown), indicating that temporal sampling had little impact on the estimation of allele frequencies for those locations. For all other locations, all specimens were collected exclusively (or almost exclusively) from one time period (i.e. within three years of each other). The finding of little genetic structure across the archipelago for all markers across samples collected during different time periods suggests that temporal sampling had little impact on allele frequency estimates.

**Figure 4:**
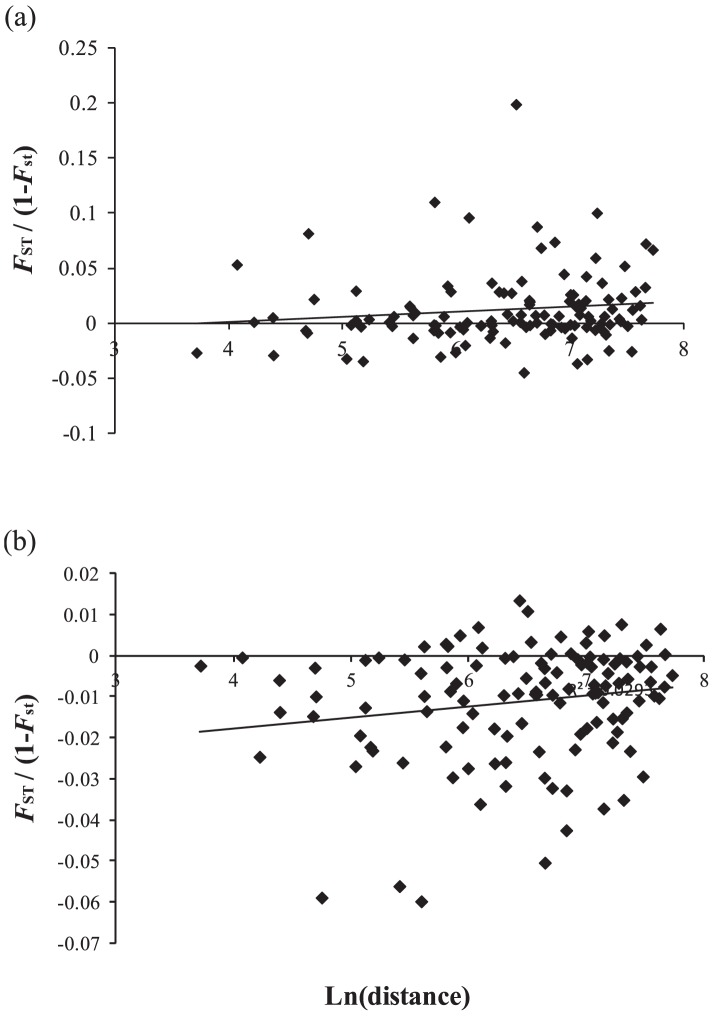
Correlation between genetic distance (pairwise *Φ*_ST_ or *F*_ST_) and geographic distance between sample sites for (a) mtDNA cytochrome *b* (*P* = 0.044, *r* = 0.126) and (b) microsatellites (*P* = 0.042, *r* = 0.171) for *Etelis coruscans*. Correlations between genetic and geographic distance were non-significant for both marker types for *Etelis marshi*.

For *E. coruscans*, genetic divergence was significantly correlated with geographic distance for both mtDNA (*P* = 0.044) and microsatellites (*P* = 0.042), although the correlation coefficient (*r*) was low for both marker types (mtDNA *r* = 0.126; microsatellites *r* = 0.171) (Figure 4). For *E. marshi*, genetic divergence was not significantly correlated with geographic distance for mtDNA (*P* = 1.000) or microsatellites (*P* = 0.833).

All values of Fu's *F*_s_ and most values of Tajima's *D* were negative and significant (*P*<0.05) for all sites for both species; locations which did not have a significant Tajima's *D* included Pearl & Hermes, Twin Banks, and Kaho'olawe for *E. coruscans* (Table 6), and Gardner for *E. marshi* (Table 7). Coalescence analyses of the mtDNA data provided estimates for age of Hawaiian populations ranging from 5,100 to 9,180 years for *E. coruscans* (Table 6); and ranging from 344,000 to 378,000 years for all locations for *E. marshi* except Gardner, which had an age estimate of 8,030 (Table 7). Initial female effective population sizes ranged from 0 to 35.9 for *E. coruscans* and were 0 at all sites for *E. marshi*; these low values indicate a very small population size prior to population expansion. For *E. coruscans*, current female effective population sizes at all locations reached the maximum possible value in ARLEQUIN, which was 99,999 (reported in Table 6 as ∞); for *E. marshi*, current female effective population sizes ranged from 2,000 to 7,850 for all locations except Gardner, which had the maximum possible value in ARLEQUIN.

**Table 6:** Tajima's *D* values, Fu's *F*_S_ values, and mismatch distribution parameter estimates calculated using mtDNA cyt*b* sequences for *Etelis coruscans*. *t*: coalescent time; ⊖_0_ (*N*_f*t = 0*_): initial female effective population size; ⊖_1_(*N*_f*t = 1*_): post-expansion female effective population size.

	Tajima's *D*	Fu's *F*_S_	*t* (years)	⊖_0_ (*N*_f*t = 0*_)	⊖_1_(*N*_f*t = 1*_)
Pearl & Hermes	−0.90	−1.94*	1.1	0.00352	∞
			(5,610)	(35.9)	(NA)
Lisianski	−1.63*	−5.65*	1.3	0	∞
			(6,630)	(0)	(NA)
Maro Reef	−1.77*	−6.30*	1	0	∞
			(5,100)	(0)	(NA)
Raita	−1.83*	−5.02*	1.2	0	∞
			(6,120)	(0)	(NA)
Gardner	−2.11*	−23.1*	1.4	0.00176	∞
			(7,140)	(18)	(NA)
St. Rogatien	−1.83*	−7.48*	1.8	0	∞
			(9,180)	(0)	(NA)
Brooks Banks	−1.78*	−7.29*	1.1	0	∞
			(5,610)	(0)	(NA)
Twin Banks	−0.97	−2.58*	1.3	0.00176	∞
			(6,630)	(18)	(NA)
Nihoa	−1.83*	−6.79*	1.4	0	∞
			(7,140)	(0)	(NA)
Ni'ihau	−1.51*	−5.74*	1.3	0.00176	∞
			(6,630)	(18)	(NA)
Kaua'i	−1.78*	−7.80*	1.3	0	∞
			(6,630)	(0)	(NA)
O'ahu	−1.82*	−8.95*	1.2	0	∞
			(6,120)	(0)	(NA)
Moloka'i	−2.11*	−24.9*	1.4	0	∞
			(7,140)	(0)	(NA)
Maui	−1.57*	−6.78*	1.2	0	∞
			(6,120)	(0)	(NA)
Kaho'olawe	−0.83	−1.93*	1.4	0	∞
			(7,140)	(0)	(NA)
Big Island	−2.10*	−18.5*	1.3	0	∞
			(6,630)	(0)	(NA)

Estimates of ⊖_1_ yielded the maximum allowable value (99,999, here indicated by ∞), so that the calculation of *N_ft = 1_* was not possible (NA).

*P<0.05.

**Table 7:** Tajima's *D* values, Fu's *F*_S_ values, and mismatch distribution parameter estimates calculated using mtDNA cyt*b* sequences for *Etelis marshi*. *t*: coalescent time; ⊖_0_ (*N*_f*t = 0*_): initial female effective population size; ⊖_1_(*N*_f*t = 1*_): post-expansion female effective population size.

	Tajima's *D*	Fu's *F*_S_	*t* (age)	⊖_0_ (*N*_f*t = 0*_)	⊖_1_(*N*_f*t = 1*_)
Northampton	−1.68*	−5.48*	3.0	0	0.3165
			(344,000)	(0)	(3,630)
Maro Reef	−2.09*	−5.85*	3.0	0	0.2620
			(344,000)	(0)	(3,000)
Raita	−2.11*	−4.73*	3.0	0	0.3214
			(344,000)	(0)	(3,690)
Gardner	−1.39	−3.18*	0.7	0	∞
			(8,030)	(0)	(NA)
St. Rogatien	−1.73*	−3.44*	3.0	0	0.2425
			(344,000)	(0)	(2,780)
Brooks Banks	−1.67*	0.058	3.3	0	0.6843
			(378,000)	(0)	(7,850)
Necker	−2.05*	−7.03*	3.0	0	0.3141
			(344,000)	(0)	(3,600)
Twin Banks	−2.13*	−8.89*	3.0	0	0.3190
			(344,000)	(0)	(3,660)
Ni'ihau	−1.86*	−7.16*	3.0	0	0.2921
			(344,000)	(0)	(3,350)
Kaua'i	−1.45*	−2.84*	3.0	0	0.2872
			(344,000)	(0)	(3,290)
O'ahu	−1.56*	−2.92*	3.0	0	0.19487
			(344,000)	(0)	(2,230)
Moloka'i	−2.07*	−6.82*	3.0	0	0.17412
			(344,000)	(0)	(2,000)
Maui	−1.92*	−4.50*	3.0	0	0.3360
			(344,000)	(0)	(3,850)
Big Island	−1.90*	−7.50*	3.0	0	0.3165
			(344,000)	(0)	(3,630)

Some estimates of ⊖_1_ yielded the maximum allowable value (99,999, here indicated by ∞), so that the calculation of *N_ft = 1_* was not possible (NA).

*P<0.05.

## Discussion

Population genetic analyses across the Hawaiian Archipelago for the deepwater fishes *E. coruscans* and *E. marshi* revealed similar patterns of genetic structure, and this structure is concordant with the submesophotic fishes surveyed thus far in Hawai'i, *H. quernus* and *P. filamentosus* [2], [3], [27]. Each of these four species exhibited little or no genetic structure across the archipelago, with the exception of genetically divergent populations occurring in the center of the archipelago (e.g., Gardner, St. Rogatien, Brooks Banks, Necker). Additionally, the highest mtDNA diversity occurred at a location in the mid-archipelago for three of these four species (at Gardner and Brooks Banks for *H. quernus* and *E. marshi*; at St. Rogatien for *E. coruscans*). *E. coruscans* was the only species with evidence of a genetically divergent population outside of the mid-archipelago (Kaua'i).

Coalescence analyses of mtDNA data indicate that the Hawaiian population of *E. coruscans* is much younger than sympatric *E. marshi* (<10,000 years vs >300,000 years; Table 6, Table 7). This may be due to a recent bottleneck in *E. coruscans*. A more intriguing possibility, that this species is a recent arrival to Hawaii, awaits testing with specimens from outside the archipelago. Current female effective population sizes for *E. coruscans* reached the maximum value in ARLEQUIN, >99,999 (reported in Table 6 as ∞) as compared to <10,000 for *E. marshi*. Hence coalescence analyses, while offering only first order approximations of these population parameters, indicate that the *E. coruscans* population in Hawaii is younger and larger than *E. marshi* over recent evolutionary history. The exception to both trends is *E. marshi* at Gardner in the mid-archipelago, which had an age estimate of only 8,030 (Table 7), and an estimated female effective population size of the maximum possible value in ARLEQUIN, >99,999. We provisionally attribute this finding to violations of coalescence assumptions at a location receiving larval input from an unsampled, genetically distinct location outside the Hawaiian Archipelago (see below), although this *ad hoc* interpretation is subject to further scrutiny.

Life history traits that might influence the population structure of these four species (spawning time, larval duration, adult vagility, etc.) are similar but not identical across species. The most dispersive life stage for these fishes is probably the larval stage. Spawning timing may influence dispersal of pelagic eggs and larvae due to seasonality of oceanographic currents in the Hawaiian Archipelago [5]. *H. quernus*, *P. filamentosus*, *E. coruscans*, and *E. marshi* are all broadcast spawners, and all have similar spawning periods (approximately six months), although *P. filamentosus* has a slightly longer spawning period (approximately ten months) [16]. Peak spawning activity occurs during the summer for each of these species except *H. quernus*, which peaks in the spring.

The length of time that larvae are in the water column (the pelagic larval duration, or PLD) also likely influences dispersal and genetic connectivity for these four deepwater fish [50]. Eggs of *H. quernus*, *P. filamentosus*, *E. coruscans*, and *E. marshi* remain in the water column no more than 48 hours before hatching [16]. Biophysical modeling indicates that these eggs could travel up to 50 km (A. Vaz unpublished data). The length of time the larvae remain in the water column after hatching is not well known. For *H. quernus*, the PLD is estimated to be 35–45 days (R. Nichols and E. DeMartini pers. comm.). PLD is not known for *P. filamentosus*, *E. coruscans*, or *E. marshi*, but eteline lutjanids typically have longer PLDs than *H. quernus* [51], [52]. For *P. filamentosus*, a captive rearing study suggests a potential PLD estimate of 52–120 days, based on observations of larvae associating with the bottom as early as 52 days and the completion of larval metamorphosis by 120 days after hatching [16].

Fishes that are benthic as adults (*E. marshi* and *H. quernus*) might be expected to have more structure than fishes that are epibenthic as adults (*E. coruscans* and *P. filamentosus*). Contrary to this expectation, neither of the bottom-dwelling species showed higher structure in pairwise *F*_ST_ comparisons. The significant structure at mid-archipelago (based on microsatellites) was *F*_ST_ = 0.008–0.010 and *F*_ST_ 0.008–0.013 for the bottom dwellers, and *F*_ST_ = 0.011–0.013 and *F*_ST_ = 0.004–0.066 for the pelagic roamers. Further, significant isolation by distance was detected in one bottom dweller (*H. quernus*) and one pelagic feeder (*E. coruscans*), providing no clear pattern based on habitat preference. Despite modest differences in spawning time, PLD, and habitat use, Hawaiian submesophotic fishes have concordant population structure that is not shared with fishes that inhabit adjacent shallow habitat.

### Connectivity with Johnston Atoll

In previous genetic studies of submesophotic reef fishes (*H. quernus* and *P. filamentosus*) in the Hawaiian Archipelago, the authors hypothesized that the pattern of population structure and high diversity in the mid-archipelago is driven by connectivity with Johnston Atoll [2]–[4]. Johnston Atoll is the nearest landmass to the Hawaiian Archipelago, with the closest Hawaiian island/atoll being French Frigate Shoals, in the mid-archipelago between Brooks Banks and Necker, 865 km north-northeast of Johnston (Figure 1). Several studies have found connectivity between Johnston and the Hawaiian Archipelago for a broad taxonomic range of fishes and invertebrates (e.g., [8], [11], [14], [15], [53]). In some cases, these species exhibit greater genetic connectivity between Johnston and the mid-archipelago than between Johnston and other parts of the archipelago (e.g., [14], [15], [53]). In addition, biogeographic studies support a dispersal corridor between Johnston and the mid-archipelago. Based on marine faunal affinities, Johnston is regarded as part of the Hawaiian biogeographic province [54]–[59]. Finally, a biophysical model of oceanic dispersal indicates two dispersal corridors from Johnston Atoll to the Hawaiian Archipelago for marine species with PLDs greater than 40 days, one centered at French Frigate Shoals, and the other at Kaua'i [5]. The latter is notable because Kaua'i is the only location that was strongly differentiated in STRUCTURE analysis of *E. coruscans*. In addition, biophysical modeling of larval dispersal for the submesophotic fish *H. quernus* indicated connectivity between Johnston and Gardner, but not other locations in Hawai'i [3].

Unfortunately none of the genetic studies on the submesophotic reef fishes of Hawai'i have included specimens from Johnston Atoll, and therefore the hypothesis of genetic connectivity between Hawai'i and Johnston has not been directly tested. The PLDs of *E. coruscans* and *E. marshi* are likely greater than 45 days, which is sufficient to maintain connectivity between the mid-archipelago and Johnston Atoll according to the biophysical models [5]. Additionally, each of these species has a wide-spread distribution across the Indo-Pacific, indicating high dispersal capabilities. Studies are currently underway to examine population genetic structure across the ranges of these species to get a greater understanding of dispersal patterns.

### Comparing genetic structure revealed by different markers and analysis types

Although the pattern of genetic structure and high diversity in the mid-archipelago was consistent across the four submesophotic reef fishes in the Hawaiian Archipelago, not all genetic markers or analyses consistently revealed these patterns within species. For example, mtDNA analyses of *H. quernus*, *E. coruscans*, and *E. marshi* revealed a greater number of significant pairwise divergence values than did microsatellite analyses; and *P. filamentosus* exhibited the opposite pattern. Obtaining different results for mtDNA and microsatellite *F*_ST_ analyses is common, and is usually attributed to differences in mutation rates, modes of inheritance, and/or levels of power between marker types [60]–[64]. Obtaining different results for different types of analyses that use the same marker is common as well (reviewed in [60], [61]), and this was observed in some cases for the fishes studied here. For example, microsatellite Bayesian clustering analyses sometimes indicated genetically divergent populations that were not detected with microsatellite pairwise *F*_ST_ analyses, and vice versa (e.g. Bayesian clustering analyses indicated Kaua'i was divergent from other locations for *E. coruscans*, but pairwise *F*_ST_ analyses did not). These subtle differences in genetic structure for different markers and analyses and the overall pattern of weak genetic structure across the archipelago for all four species are consistent with a scenario in which migration across the archipelago is around the level at which genetic methods lose power to detect restrictions in gene flow. Genetic methods lose power at levels of gene flow that are still low enough to maintain demographically independent populations; this is especially true when effective population sizes are large, as in many marine fish and invertebrate populations [65], [66]. The idea that demographically relevant population structure occurs within the Hawaiian Archipelago is also supported by the presence of significant genetic isolation by distance for two of the deepwater species (*H. quernus*, [67]; and *E. coruscans*). Therefore the structure detected for deepwater fish using genetic methods is likely biologically relevant on an ecological timescale.

### Submesophotic population structure

The potential influence of unique oceanographic features of the submesophotic habitat on population genetic structure is largely unknown. Species inhabiting these environments may be less dispersive if larvae are not subject to the high-energy environment of surface waters [68]. A previous study of a submesophotic snapper (*Pristipimoides multidens*) in Indonesia and northern Australia supported this idea, with surprisingly strong genetic structure found over short geographic distances (e.g. genetically divergent groups separated by as little as 191 km) [69]. In contrast, the population structure observed in the four submesophotic fishes is typical of shallow-water members of the same taxonomic families [10], [70] with the exception of the mid-archipelago structure that so far is unique to submesophotic fishes. Indeed, it is not certain that spawned eggs or larvae remain at submesophotic depths, and so this expectation may not be valid. Another possibility is that deeper communities may be less susceptible to the disruption that shallow communities experience during sea level drops associated with glaciations [68], [71], [72]. During the last glacial maximum, sea levels dropped at least 130 m below contemporary levels [73]. Collectively the four fishes evaluated here range down to ∼330 m, and adult *Etelis* specimens have been documented below that depth; this would seem to be ample buffer against disturbance in the upper 130 m. Furthermore, the primary habitat of these species consists of rocky substrate, not living coral, and therefore the abundance of suitable habitat may not be substantially influenced by sea level changes. Nevertheless, these fish show the short coalescence times that are typical of shallow reef populations ([10], [70] for examples): ∼6,000 years for *E. coruscans* and ∼344,000 years for *E. marshi*. While this topic requires further investigation, especially for the coral reef fauna at mesophotic depths, the evidence here indicates that submesophotic fishes experience the same type of episodic crashes as confamilial species in shallow depths.

### Conservation & Management

*E. coruscans*, *E. marshi*, *H. quernus*, and *P. filamentosus* comprise the majority of a valuable deepwater fishery in Hawai'i [74]–[76] and they have experienced overfishing in recent years [48]. The Northwestern Hawaiian Islands (Nihoa to Kure) have been off-limits to the fishery since 2010 due to the designation of this region as the Papahānaumokuākea Marine National Monument. However, the presence of fishing pressure in the Main Hawaiian Islands necessitates an understanding of stock structure across the archipelago for the effective management of this fishery. Population genetic analyses of these four deepwater fishes point to the mid-archipelago as a genetically diverse region possibly with larval supplement from an external source. Genetic analyses reveal high levels of connectivity across the rest of the archipelago, with the possible exception of a genetically divergent population of *E. coruscans* at Kaua'i. The genetic data provide several indications that dispersal between Hawaiian Islands may be at a level low enough to create some level of demographic independence, but high enough to prevent genetic divergence between these regions. Other methods for measuring demographic connectivity, such as tagging studies and bio-physical modeling of larval dispersal, will be an important complement to these genetic data for the assessment of stock structure.

In the realm of dispersive and migratory marine species, there are many cases in which genetically divergent groups are distributed in complex geographic configurations, and these cases often necessitate complex management strategies. For example, both sea turtles and salmon have genetically differentiated stocks at spawning habitats, but these stocks are diffuse and thoroughly mixed in oceanic feeding habitats [77], [78]. For marine species with pelagic larvae, an increasing number of studies are finding evidence that complex genetic structure depends more on oceanographic currents or habitat distribution than on simple geographic distance [79], [80]. The Hawaiian submesophotic fishes also appear to have geographically complex population genetic structure that is not defined solely by geographic distance, because locations in the mid-archipelago are genetically distinct from nearby locations. If future studies find this genetic divergence to be driven by dispersal from Johnston Atoll, then larval supplement to the local Hawaiian stock from Johnston Atoll may need to be incorporated into management strategies.

### Conclusions

Population genetic studies have revealed high variability in patterns of genetic structure across the Hawaiian Archipelago for shallow marine species, even for species with similar life history characters. In contrast, all four submesophotic fishes surveyed to date showed concordant patterns of genetic structure, with dispersal across the entire archipelago, yet genetically divergent populations occurring in the mid-archipelago. Genetic divergence at the mid-archipelago may be driven by connectivity with Johnston Atoll, located outside of the Hawaiian Archipelago. Therefore management strategies may need to consider the impact of larval supply from outside the Hawaiian Archipelago, a supplement that has yet to be quantified. Our results also indicate that submesophotic populations may be as dispersive as their shallow water counterparts, and may have been equally unstable over geological time.

## Supporting Information

Table S1 **Specimen ID, sampling location, microsatellite allele lengths (bp), and cyt** ***b*** **GenBank Accession numbers for Etelis coruscans specimens.** Missing data coded by “0”.
(TXT)

Table S2 **Specimen ID, sampling location, microsatellite allele lengths (bp), and cyt** ***b*** **GenBank Accession numbers for Etelis marshi specimens.** Missing data coded by “0”.
(TXT)
